# Supplementary material for: Asynchronous magnetic resonance elastography: Shear wave speed reconstruction using noise correlation of incoherent waves
Source: Magn Reson Med. 2022 Oct 27;89(3):990–1001. doi: 10.1002/mrm.29502 (PMC9792433; doi:10.1002/mrm.29502)
Supplement: Supplementary file 1 — DATA S1 MATLAB code used to generate the numerical simulations of Figure 1 [file MRM-89-990-s002.zip › k-Wave/helpfiles/getAlphaFilter.html]

getAlphaFilter :: Functions (k-Wave)


# getAlphaFilter

Create filter for `medium.alpha_filter`.

## Syntax

```
alpha_filter = getAlphaFilter(kgrid, medium, filter_cutoff)
alpha_filter = getAlphaFilter(kgrid, medium, filter_cutoff, taper_ratio)
```

## Description

`getAlphaFilter` uses `getWin` to create a Tukey window via rotation to pass to the `medium.alpha_filter` input field of the first order simulation functions (`kspaceFirstOrder1D`, `kspaceFirstOrder2D`, and `kspaceFirstOrder3D`). This parameter is used to regularise time reversal image reconstruction when absorption compensation is included.

## Inputs

|  |  |
| --- | --- |
| `kgrid` | k-Wave grid object returned by kWaveGrid |
| `medium` | k-Wave medium structure |
| `filter_cutoff` | alpha\_filter cutoff frequency [Hz], where  `filter_cutoff = [f_all]` in 1-D `filter_cutoff = [f_all]` or `[f_x, f_y]` in 2-D `filter_cutoff = [f_all]` or `[f_x, f_y, f_z]` in 3-D  Any of the filter\_cutoff inputs may be set to `'max'` to set the cutoff frequency to the maximum frequency supported by the grid |

## Optional Inputs

|  |  |
| --- | --- |
| `taper_ratio` | taper ratio for Tukey Window (default = 0.5) |

## Outputs

|  |  |
| --- | --- |
| `alpha_filter` | filter |

## Examples

- Image Reconstruction With Compensation For Acoustic Absorption

## See Also

`getWin`, `kspaceFirstOrder1D`, `kspaceFirstOrder2D`, `kspaceFirstOrder3D`
